# Perineal hernia repair with a combined abdominoperineal approach with biologic mesh placement and peritoneal flap reconstruction

**DOI:** 10.1093/jscr/rjae695

**Published:** 2024-12-10

**Authors:** Gabrielle Massé, M Al Khaldi, F Schwenter, E Coeugniet, H Sebajang

**Affiliations:** Digestive Surgery Service, Department of Surgery, Centre Hospitalier de l’Université de Montréal (CHUM), 1000 rue St-Denis, Montreal, QC H2X 0C1, Canada; Digestive Surgery Service, Department of Surgery, Centre Hospitalier de l’Université de Montréal (CHUM), 1000 rue St-Denis, Montreal, QC H2X 0C1, Canada; Digestive Surgery Service, Department of Surgery, Centre Hospitalier de l’Université de Montréal (CHUM), 1000 rue St-Denis, Montreal, QC H2X 0C1, Canada; Plastic Surgery Service, Department of Surgery, Centre Hospitalier de l’Université de Montréal (CHUM), 1000 rue St-Denis, Montreal, QC H2X 0C1, Canada; Digestive Surgery Service, Department of Surgery, Centre Hospitalier de l’Université de Montréal (CHUM), 1000 rue St-Denis, Montreal, QC H2X 0C1, Canada

**Keywords:** pelvic floor reconstruction, abdominoperineal approach, peritoneal flap, combined approach, perineal hernia

## Abstract

Perineal hernias occur rarely following abdominoperineal resections. No standardized surgical approach exists for treating PH. We herein present the case of a large, symptomatic PH that was repaired with a combined abdominal and perineal approach, with peritoneal flap reconstruction of the pelvic floor and placement of a biological mesh. The patient has not recurred after 3 years of follow-up. In conclusion, despite the lack of a standardized approach for tackling perineal hernias, a combined one with peritoneal flap reconstruction can be successfully used.

## Introduction

Perineal hernias (PH) manifest as the protrusion of intraabdominal contents through a defect in the pelvic floor. They can be congenital or secondary to prolonged ascites [1], constipation [2], trauma, or pelvic surgery, including prostatectomy, abdominoperineal resection (APR), and pelvic exenteration (PE) [3]. Patients may exhibit pelvic bulging, discomfort when sitting or standing [4], incarceration, bowel obstruction, and less commonly urinary dysfunction [5].

Management is conservative, with surgical intervention considered in rare cases. Surgical options encompass perineal and abdominal techniques [1], however, no standardized surgical approach exists. In this article, we present a patient with PH who underwent surgical management using a combined abdominoperineal approach, with closure of the pelvic floor defect with a peritoneal flap and the placement of a biologic mesh.

## Case presentation

**Figure 1:**
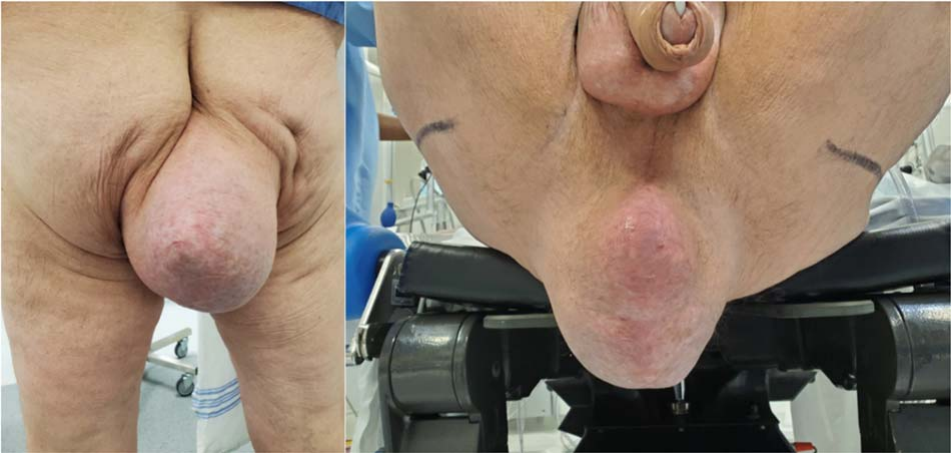
A perianal bulge can be appreciated on this picture. The picture was given with the patient’s consent.

A 61-year-old man with a past surgical history of APR for low rectal cancer presented with a progressively enlarging bulge in the perineal region several months after his surgery. Upon physical examination, a substantial PH was observed, characterized by the thinning of the overlying skin (Fig. 1).

**Figure 2:**
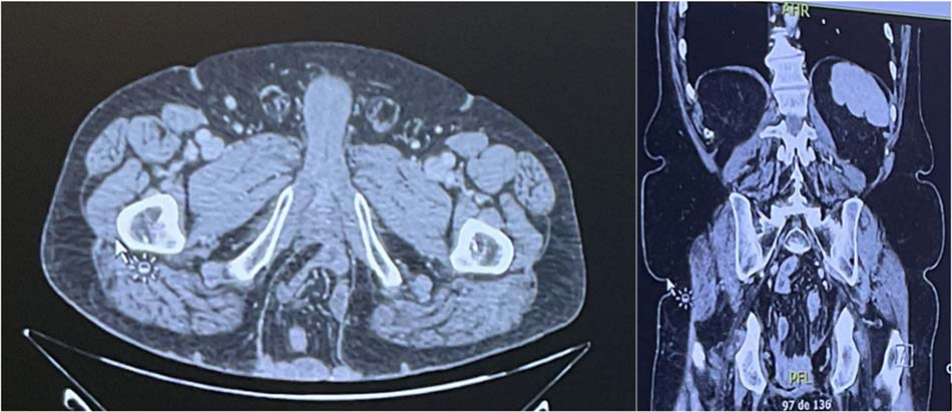
CT scan revealing a PH of 9.5 × 8.7 × 15 cm, containing small bowel.

A computed tomography (CT) scan showed a PH of 9.5 × 8.7 × 15 cm, containing small bowel, with no sign of incarceration (Fig. 2). Given the symptomatic nature of the PH, a combined surgical procedure in collaboration with our plastic surgery team was planned.

The procedure was performed under general anesthesia, with the patient in lithotomy position. A midline laparotomy incision was performed. The small bowel was carefully mobilized from the pelvic cavity. The plastic surgery team then performed a skin incision on the perineum followed by the separation of the skin from the hernia sac. Excess skin and part of the hernia sac were resected. The remaining hernia sac was pushed back into the pelvic cavity. The hernia defect was then closed on the perineal side. An acellular dermal matrix (AlloDerm) was then secured in place, anchoring it to the ischium anteriorly, ischial ramus laterally, ischial tuberosity postero-laterally, and posteriorly to the coccyx. The dead space was then closed in three separate planes followed by skin closure. Two closed-suction drains were placed above and below the matrix.

On the abdominal side, the remaining hernia sac was closed in two planes. Bilateral peritoneal flaps were dissected off the ureters and sutured together in the pelvic cavity, further separating it from the perineum and preventing the small bowel from herniating through. The abdominal wall was finally closed primarily.

**Figure 3:**
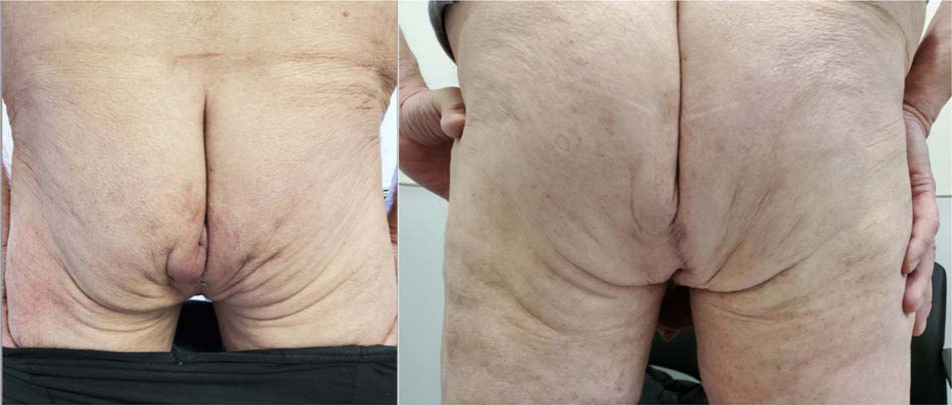
The repaired perineum at 1 month (A) and completely healed wound at 4 months (B).

On follow-up, the patient experienced a satisfactory postoperative recovery (Fig. 3) and showed no signs of recurrence after 3 years of follow-up.

## Discussion

Over the last few years, the number of reported cases of PH has been rising, pointing to a renewed interest in this entity possibly due to an increased incidence of PH [5–7]. The recent advancements in colorectal cancer may play a role in this phenomenon. Modifications in the APR technique [6, 8], with the conventional coning of the specimen being replaced by ELAPE, with a cylindrical specimen resection, increase the risk of PH due to the more extensive pelvic resection [7–9]. Although ELAPE reduces the number of positive margins [10] and improves the oncological outcome, the incidence of postoperative PH ranges from 1% to 13% with the conventional technique and 26% with ELAPE [9]. The recent use of neoadjuvant chemotherapy and radiotherapy [5] also plays a role in PH occurrence by increasing the risk of wound complications [7]. The patient presented in this report had received neoadjuvant radiation therapy prior to APR, which could have contributed to the development of his PH.

The indications for surgical reduction are symptomatic or large-sized hernias [8]. The operative approaches include a transabdominal (laparoscopic or open), a perineal, or a combined abdominoperineal approach [9]. The abdominal route permits a better visualization of the hernia content and mobilization of the bowel [5]. However, it is associated with a relatively longer postoperative recovery time [11] and an increased overall rate of complications [12]. The perineal route allows a better access for mesh placement and repair of the perineal defect. It is also associated with a significantly shorter length of stay [7, 12]. Recurrence rate for each approach varies in the literature. However, the abdominoperineal approach has the lowest recurrence rate [5, 9].

Options for perineal defect closure include primary closure, synthetic or biological mesh [8], and myocutaneous flaps. Primary repair, mesh repair, and combined mesh-flap repair have a similar recurrence rate, while flap alone has a significantly higher recurrence rate [5]. The use of a mesh significantly reduces the risk of abdominal wall hernia [13] but presents an inherent risk of adhesions, erosion, and fistulas since the small bowel lies directly on the mesh, without a layer of perineum underneath [8]. Biological meshes are associated with a reduced risk of bowel erosion [5], infection, and adhesion, but an increased reported pain during recovery [13]. Synthetic meshes have a lower recurrence rate than biological mesh [9].

In our case, an abdominoperineal approach was used with a combination of primary closure of the hernia defect and placement of a biological mesh. We also reconstructed the pelvic floor with bilateral peritoneal flaps to isolate the intraabdominal content from the perineum and further mitigate the risk of herniation and bowel fistula formation.

## Conclusion

In this report, we present the case of a large PH that was treated by an abdominoperineal approach with a biologic mesh and peritoneal reconstruction. The patient had no recurrence at 3-year follow-up. A combined approach with a biologic mesh is a viable approach for PH repair. A peritoneal flap reconstruction as a potential means to reduce overall morbidity and further reduce the risk of herniation could be added to enhance perineal repair.
